# Comparison of the TightRope system versus hook plate in acute acromioclavicular joint dislocations: a retrospective analysis

**DOI:** 10.1038/s41598-021-90989-8

**Published:** 2021-05-31

**Authors:** Guangsi Shen, Shengxuan Sun, Chengyang Tang, Ye Xie, Liubing Li, Wei Xu, Youjia Xu, Haibin Zhou

**Affiliations:** Department of Orthopaedics, The Second Affiliated Hospital of Soochow University, 1055 Sanxiang Road, Suzhou, 215004 Jiangsu People’s Republic of China

**Keywords:** Trauma, Bone, Ligaments

## Abstract

This study compared the results of the minimally invasive coracoclavicular (CC) fixation with a single TightRope (MITR) procedure and the hook plate (HP) procedure for acute acromioclavicular (AC) joint dislocation treatment. Sixteen patients with a mean age of 44.9 ± 11 years were treated with the MITR procedure. Nineteen patients with a mean age of 40.2 ± 8.7 years were treated using the HP procedure. Clinical outcomes were evaluated with the Visual Analog Scale (VAS) for pain, Constant–Murley Score (CMS), and University of California at Los Angeles (UCLA) Shoulder score. Vertical displacement of the clavicle with reference to the height of the acromion was measured in standard anteroposterior radiographs. The mean follow-up was 27 months in the MITR group and 30 months in the HP group. No statistically significant differences were found between the MITR group and the HR group in terms of VAS score (0.4 ± 0.6 vs 0.7 ± 0.6, P = 0.138), UCLA Shoulder score (33.9 ± 2.5 vs 33.7 ± 1.5, P = 0.843), or CMS (95.7 ± 7.3 vs 93.7 ± 6.6, P = 0.400). No redislocation was identified in the HP group, while redislocation occurred in 1 of 16 (6.3%) patients in the MITR group. One patient in the HP group (5.3%) had acromial osteolysis, while no acromial osteolysis was found in the MITR group. No other adverse events, such as infections, tunnel widening, fractures, or implant-related complications, were observed. Both procedures provided satisfactory results. The HP procedure provided better reduction, while the MITR procedure provided a slightly lower tendency of pain. Long-term follow-up is needed to investigate the clinical outcomes and radiological outcomes of both groups.

## Introduction

Acromioclavicular (AC) joint dislocations are common injuries, particularly in the active population. The Rockwood classification is the most widely used method to classify for AC joint dislocations, and it is used to grade AC joint dislocations from type I to VI based on the degree and direction of distal clavicle displacement^1^. Nonsurgical treatment is usually recommended for Rockwood type I and II AC joint dislocations. Type III AC joint dislocations are further classified into type IIIA (horizontally stable) and IIIB (horizontally unstable) injuries^2^. Nonsurgical treatment is suggested for type IIIA injuries, while surgical treatment is advocated for type IIIB injuries^2^. In addition, several studies recommend operations for type III injuries in heavy manual laborers and athletes^3,4^. Surgery is usually recommended for Rockwood type IV, V and VI AC joint injuries^5–7^.

Various surgical procedures have been proposed for the management of AC dislocations, including the Weaver–Dunn procedure; coracoclavicular (CC) joint screw fixation; CC or AC joint reconstruction with autografts, allografts or synthetic grafts; tension banding; hook plate (HP) fixation; EndoButton fixation; K-wire fixation; and so on^8–15^. Although the aforementioned treatment options all play a role in the management of AC joint dislocations, no gold standard of management has yet been identified.

Currently, the HP procedure and the TightRope (TR) procedure are the two most widely used methods in the management of AC dislocations. Both techniques offer safe and effective outcomes. However, the HP procedure has some disadvantages, including pain, functional limitations, subacromial impingement, rotator cuff tears, and a second operation to remove the plate^15,16^. In a recent systematic review, Moatshe et al.^12^ reported that HP and K-wire treatment had a complication rate of 26.3% and a reoperation rate of 1.2% in both acute and chronic cases. TR procedures include single TR (STR) and double TR (DTR) procedures. STR procedures require fewer tunnels, whereas DTR procedures provide better stabilization^6^.

According to a newly published meta-analysis by Lloyd et al.^17^, the TR technique resulted in better functional outcomes and a reduced Visual Analog Scale (VAS) pain score than the HP technique, whereas other studies did not come to this conclusion^18,19^. The present study aimed to compare the clinical and radiological results of a minimally invasive CC fixation with an STR (MITR) technique and the HP technique for acute AC joint dislocation treatment. It was hypothesized that the MITR technique would provide clinical and radiological results that were comparable with those of the HP technique.

## Patients and methods

### Patient data

The study protocol was approved by the Institutional Review Board of the Second Affiliated Hospital of Soochow University. All patients provided informed consent for the procedure and study inclusion in written format. All methods were carried out in accordance with relevant guidelines and regulations.

**Figure 1:**
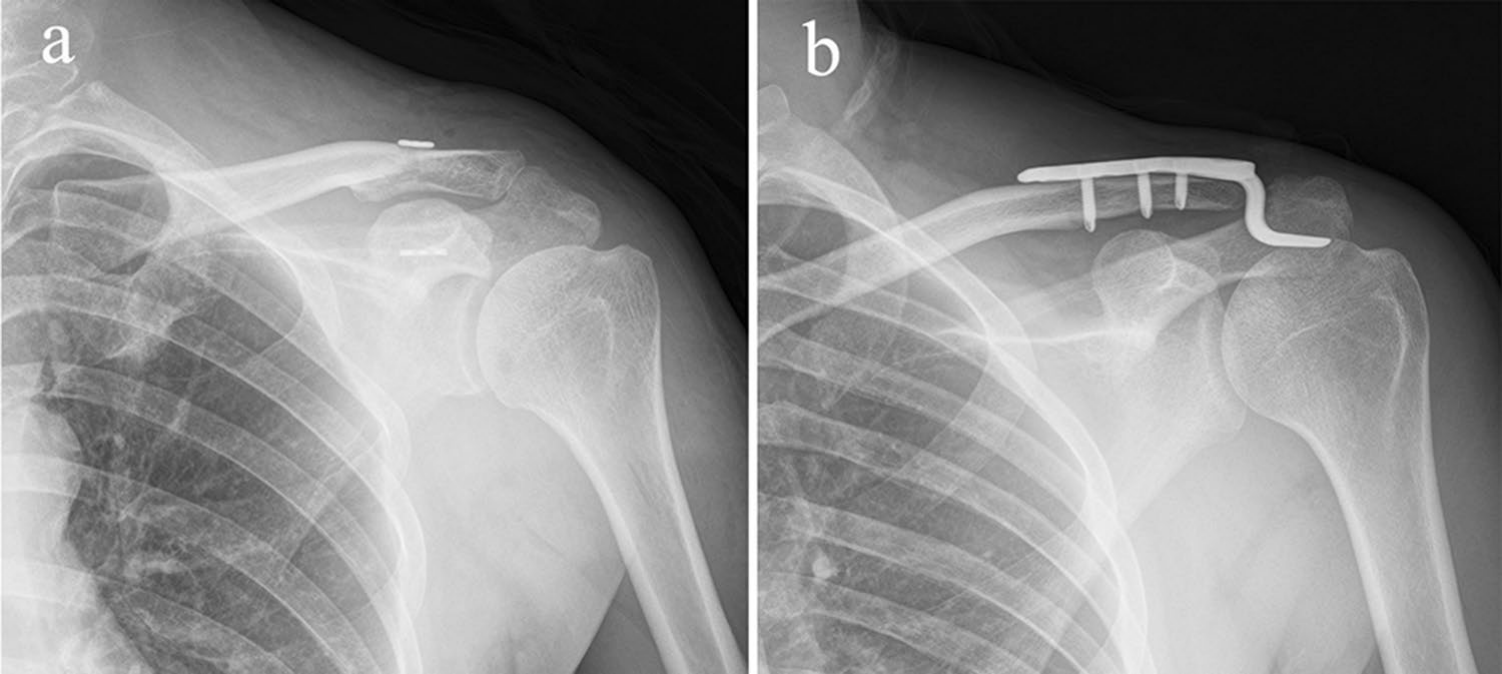
Radiographs immediately after the surgical procedures. (**a**) MITR fixation. (**b**) HP fixation.

From July 2016 to October 2018, 41 patients with acute AC joint dislocations (type III and V) were treated with surgery in our department. Eighteen patients underwent MITR (the MITR group) (Fig. 1a), and 23 patients were treated with open reduction and internal fixation using an HP (the HP group) (Fig. 1b). We conducted a retrospective study to compare MITR and HP results for acute AC joint dislocation treatment.

Inclusion criteria were as follows: (1) Rockwood type III injuries (only manual workers, overhead workers or active individuals); (2) Rockwood type V injuries; (3) acute injuries (< 3 weeks after injury); (4) age 18–60 years; (5) first-time injury of the AC joint; (6) primary treatment with HP fixation or MITR reconstruction; (7) follow-up time of at least 15 months; and (8) implants removed at the last follow-up in the HP group.

Exclusion criteria were as follows: (1) chronic injuries (> 3 weeks after injury); (2) previous AC joint injuries; (3) associated diseases that could influence assessment (e.g., cognitive impairment, psychiatric disorders, neuromuscular, and rheumatological disease); (4) abnormal shoulder function before the injury, with an inability to freely use shoulder joints in work and life (e.g., neuromuscular disorder); (5) fractures and/or dislocations in other parts of the ipsilateral limb; and (6) association with vascular or nerve injury.

### HP fixation surgical technique

The patient was placed in a beach-chair position under general anesthesia. A 6-cm transverse skin incision was made along the distal clavicle to the acromion, and the AC joint was subsequently exposed. Then, any articular cartilage debris or hematoma in the AC joint was debrided. The hook end of a prebent plate (3.5 mm, titan, DePuy Synthes, Switzerland) was inserted underneath the acromion, and the proximal end of the plate was temporarily fixed to the clavicle with a Kocher forceps. Thus, the AC joint was reduced. The AC joint reduction status, hook depth and plate position were confirmed with intraoperative fluoroscopy in two views. Then, the HP was fixed to the clavicle with at least 3 locking screws. Intraoperative fluoroscopy was used to recheck the reduction status, plate position, hook depth and screw length. The CC ligament was not exposed. We repaired the AC joint capsule with absorbable sutures.

Pain was an indication to remove the implant. If the patient had pain after surgery, the HP was removed within 3 months. If the patient did not have pain, the recommended time of removal was 6 to 8 months after the operation.

### MITR surgical technique

**Figure 2:**
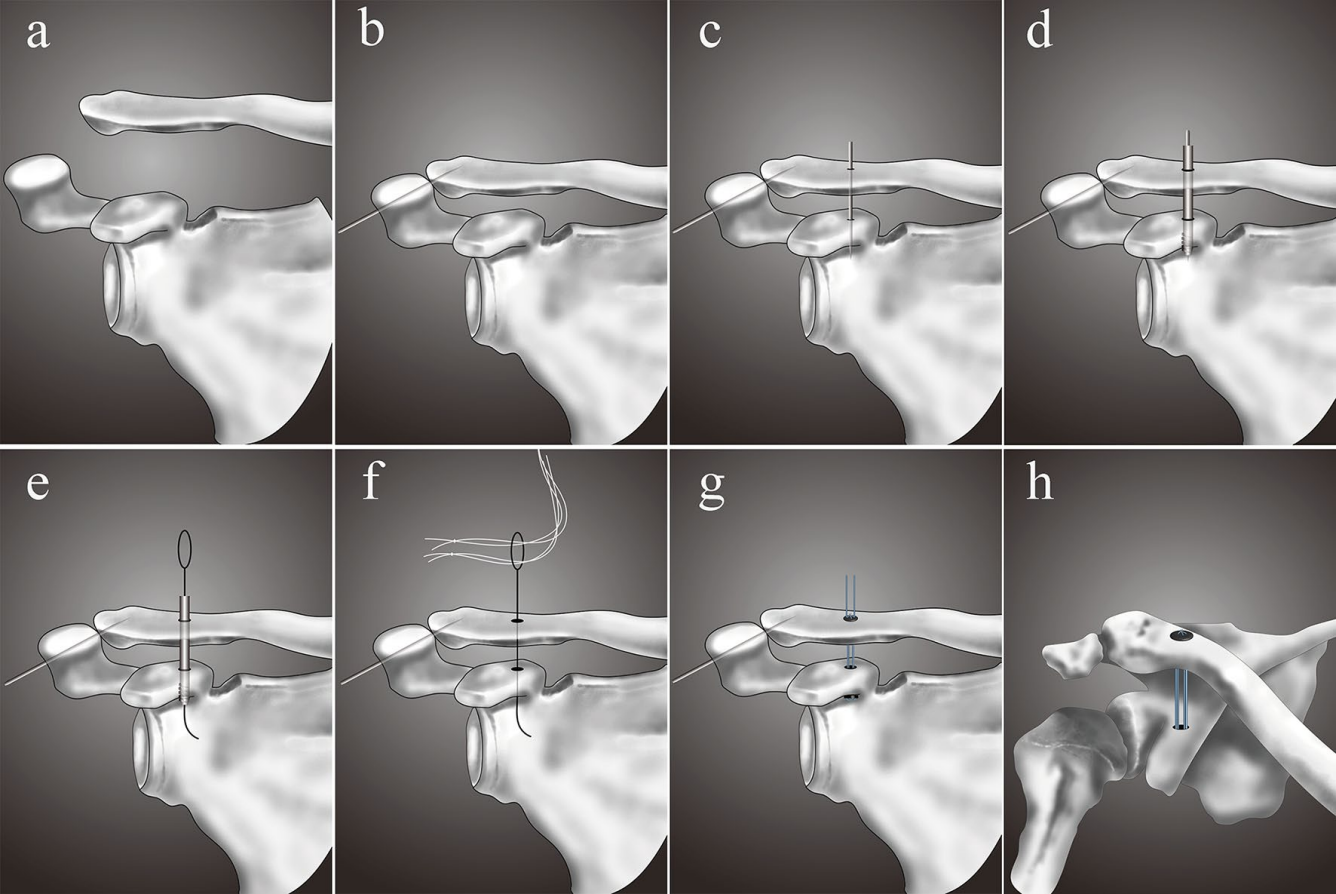
MITR operative procedure. (**a**) AC joint dislocation. (**b**) Closed reduction and fixation of AC joint dislocation with a Kirschner wire. (**c**) A 2.0-mm Kirschner wire is introduced through the clavicle and coracoid. (**d**) A 4.0-mm drill bit is drilled into the base of the coracoid. (**e**) A nitinol suture passing wire is advanced down through the cannulated drill bit. (**f**) The drill bit is removed, and the two white traction sutures are inserted into the bone tunnels through the wire loop of the nitinol suture passing wire. (**g**) The TR system is introduced, and the reduction and stabilization of the AC joint are completed. (**h**) Final frontal view of AC joint fixed with the TR system.

The patient was positioned in a beach-chair position. The AC joint was reduced and fixed with a 2.0-mm Kirschner wire. A 2-cm skin incision and a 3-cm skin incision were made to prepare the clavicle and the coracoid process, respectively. A 2.0-mm Kirschner wire was introduced from the clavicle to the coracoid. Then, a 4.0-mm drill bit was drilled from the clavicle to the base of the coracoid. A nitinol suture passing wire was advanced down through the cannulated drill bit. The drill bit was removed, and two white traction sutures were inserted into the bone tunnels through the wire loop of the nitinol suture passing wire. The oblong button was transported from the upper surface of the clavicle to the bottom of the coracoid with the two white traction sutures. The position of the oblong button was verified. Then, the round button was advanced down to the upper surface of the clavicle. Finally, the TR was fixed with sutures. The reduction was controlled with intraoperative fluoroscopy (Fig. 2).

### Rehabilitation

The rehabilitation methods were the same in the HP group and the MITR group. Pendulum exercises and fully active movement of the elbow, wrist and hand were allowed after surgery. The arm was immobilized for one month with a sling. During the first 3 weeks, passive motion was allowed up to 45° flexion and abduction. Between 3 and 6 weeks, active-assisted movements were allowed up to 60° flexion and abduction. After the 7th week, patients were allowed to move freely. Muscle-strengthening exercises were started after 10 to 12 weeks. Heavy lifting and other strenuous activities that would lead to significant downward traction of the operated arm were prohibited within 4 months postoperatively.

### Clinical evaluation

Two examiners performed follow-up evaluations (orthopedic surgeons of our department) but did not perform operations. Clinical outcomes were assessed at the last follow-up with the VAS^20^, Constant–Murley score (CMS)^21^, and University of California at Los Angeles (UCLA) Shoulder score^22^.

### Radiographic evaluation

Standard anteroposterior radiographs of the operated shoulder were obtained at the final follow-up visit to evaluate any remaining vertical AC joint instability. The vertical displacement of the clavicle with reference to the height of the acromion was measured. We considered no displacement, displacement < 50%, and displacement > 50% with reference to the height of the acromion as reduction, subluxation and redislocation, respectively.

### Statistical analysis

Statistical analyses were performed with SPSS software (ver. 20.0, IBM Inc., Chicago, USA). Continuous variables are reported as the mean ± standard deviation (SD). An independent Student’s *t* test or the Mann–Whitney *U* test was conducted to compare continuous variables with normal or non-normal distribution, respectively. The chi-square test was used to compare the categorical variables. Data are reported as the mean ± SD or median (range, minimum–maximum). Differences between the two groups were considered significant at P < 0.05.

## Results

### Study population

There were 16 patients (11 males, 5 females) in the MITR group and 19 patients (10 males, 9 females) in the HP group, with mean ages of 44.9 ± 11 years and 40.2 ± 8.7 years, respectively. The follow-up periods were 27 months (range 15–42 months) and 30 months (range 16–40 months) in the MITR group and HP group, respectively. Four patients (3 in the HP group and 1 in the MITR group) were lost to follow-up, and 2 patients (1 in the HP group and 1 in the MITR group) refused to participate. The right arm was affected in 6 and 9 patients in the MITR group and HP group, respectively. The length of time from injury to operation was 6.8 ± 3.1 days and 7.1 ± 2.2 days for the MITR group and HP group, respectively. There were no significant between-group differences in age, sex, laterality or the length of time from injury to operation (Table 1). The HPs were removed within 8 months postoperatively.

**Table 1:** Patient demographic characteristics (mean ± SD).

Parameter	MITR group	HP group	*P* values
Number of patients	16	19	N/A
Age (years)	44.9 ± 11	40.2 ± 8.7	0.171
Sex (M/F)	11/5	10/9	0.267
Left/right	10/6	10/9	0.404
Time from injury to surgery (days)	6.8 ± 3.1	7.1 ± 2.2	0.076

*M* male, *F* female, *MITR* minimally invasive coracoclavicular fixation with a single TightRope, *HP*, hook plate.

#### Clinical outcomes

Clinical outcome measures at the final follow-up are shown in Table 2. There were no significant differences in the mean VAS score, UCLA Shoulder score or CMS between the two groups.

**Table 2:** Comparison of different scores in both groups (mean ± SD).

Indexes	MITR group	HP group	*P* value
VAS	0.4 ± 0.6	0.7 ± 0.6	0.138
UCLA	33.9 ± 2.5	33.7 ± 1.5	0.843
CMS	95.7 ± 7.3	93.7 ± 6.6	0.400

*VAS* visual analog scale, *UCLA* University of California at Los Angeles Shoulder score, *CMS* Constant–Murley Score, *MITR* minimally invasive coracoclavicular fixation with a single TightRope, *HP* hook plate.

### Radiographic follow-up

In the MITR group, the anatomic reduction was finally obtained in 93.7% (15/16) of patients. In the HP group, no redislocation or subluxation was identified. There were no statistically significant differences in redislocation between the two groups (P = 0.457).

### Complications

**Figure 3:**
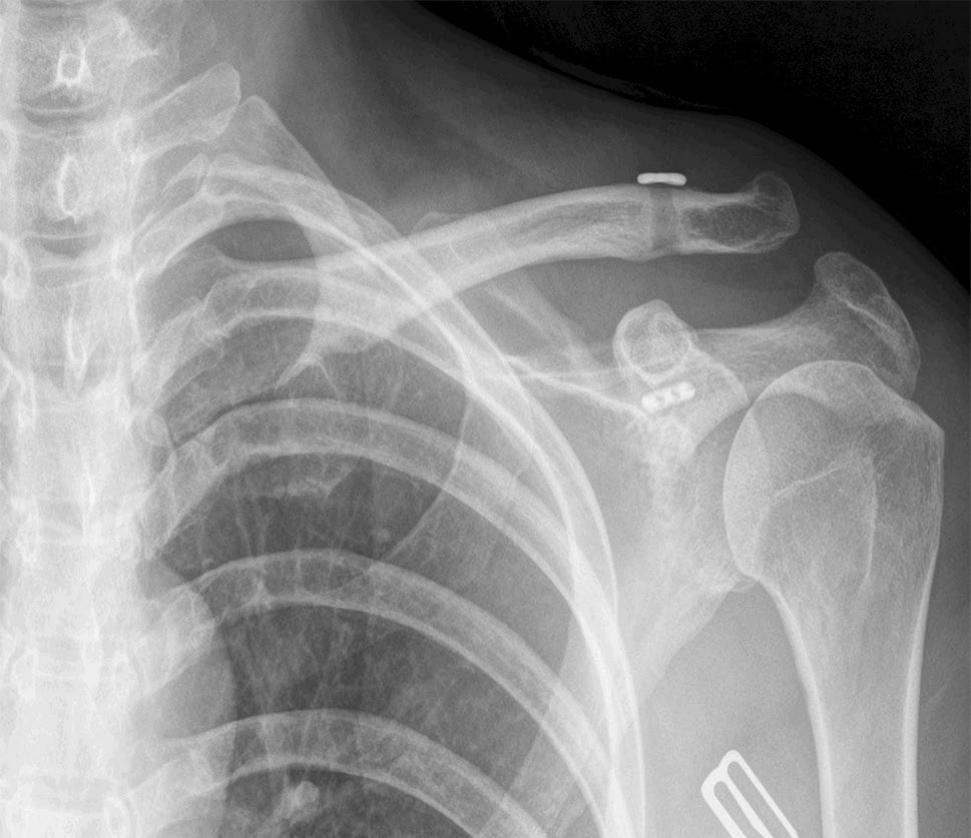
Redislocation was observed 1 day after the MITR procedure.

In the MITR group, one case of redislocation was observed 1 day after the operation, but the patient refused to undergo revision surgery (Fig. 3). In the HP group, one patient had acromial erosion. No other adverse events, such as infections, tunnel widening, fractures, or implant-related complications, were observed.

## Discussion

The most important findings of the present study are that we found that good-to-excellent functional outcomes can be obtained with both the MITR and the HP fixation to treat type III and V AC dislocations. Additionally, this study found no significant differences in pain or functional outcomes, as evaluated using the UCLA scale, VAS and CMS, at the short-term follow-up postoperatively. However, the authors acknowledged a slightly higher tendency to a VAS for HP procedure could be observed, probably due to the stress effect of the HP on the lower surface of the acromion and the second operation for implant removal.

The HP technique is popular for the treatment of AC joint dislocation. Many studies have reported satisfying results with HP fixation^16,18,23^. In a prospective study, 27 acute high-grade (Rockwood grade IV/V) AC joint dislocations were treated with the HP technique. After a 24-month follow-up, satisfactory outcomes (CMS: 90.19 ± 7.79) were obtained^16^. Jensen et al.^18^ treated 30 acute Rockwood type III/V AC joint separations using the HP technique, with a median CMS of 92.4 and a median Taft score of 10. Satisfactory outcomes resulted from the superiority of the HP technique in achieving reduction in both the vertical and horizontal planes. McConnell et al.^24^ reported that the HP technique most closely restored the biomechanics of the AC joint to normal values. However, despite these good to excellent outcomes, several studies have reported many complications of the HP technique, such as metal breakage, clavicle and coracoid fractures, subacromial erosions, rotator cuff injury, redislocation and AC degeneration^25–28^. In the present study, one patient sustained subacromial erosion. No redislocation or subluxation was observed. The mean CMS was 93.7 in the HP group, which was comparable to that reported by Arirachakaran et al.^25^.

The TR method has been used as a surgical technique for treating AC dislocations for more than 10 years^29^. Whereas the TR technique is used to reconstruct disrupted CC ligaments, the HP technique is used to reconstruct the AC joint. The most crucial advantage of the TR method is that there is no need for a second operation to remove the implant. Some studies reported better results with the TR technique than the HP technique^16,17^. Lloyd et al.^17^ conducted a systematic review including 6 clinical studies with 285 patients and reported that the suture-button (including TR) technique resulted in higher CMSs and lower VAS scores. The TR procedures include both open and arthroscopic procedures. MITR was an open TR procedure performed with minimal invasiveness, with only two incisions. Abdelrahman et al.^30^ found that the differences in outcomes, in terms of pain, function, the length of hospitalization, and CC distance, between MITR and arthroscopic procedure were statistically nonsignificant. However, they found a higher cost and longer surgical time with the arthroscopic procedure, but the arthroscopic technique had a longer learning curve. In the present study, good to excellent early clinical results, with a mean CMS of 95.7 at the final follow-up, were achieved in the MITR group. Comparable results were reported by Jensen et al.^18^ in 2014, who performed arthroscopically assisted reduction of the AC joint with the DTR technique for patients with Rockwood type III and V acute injuries.

**Figure 4:**
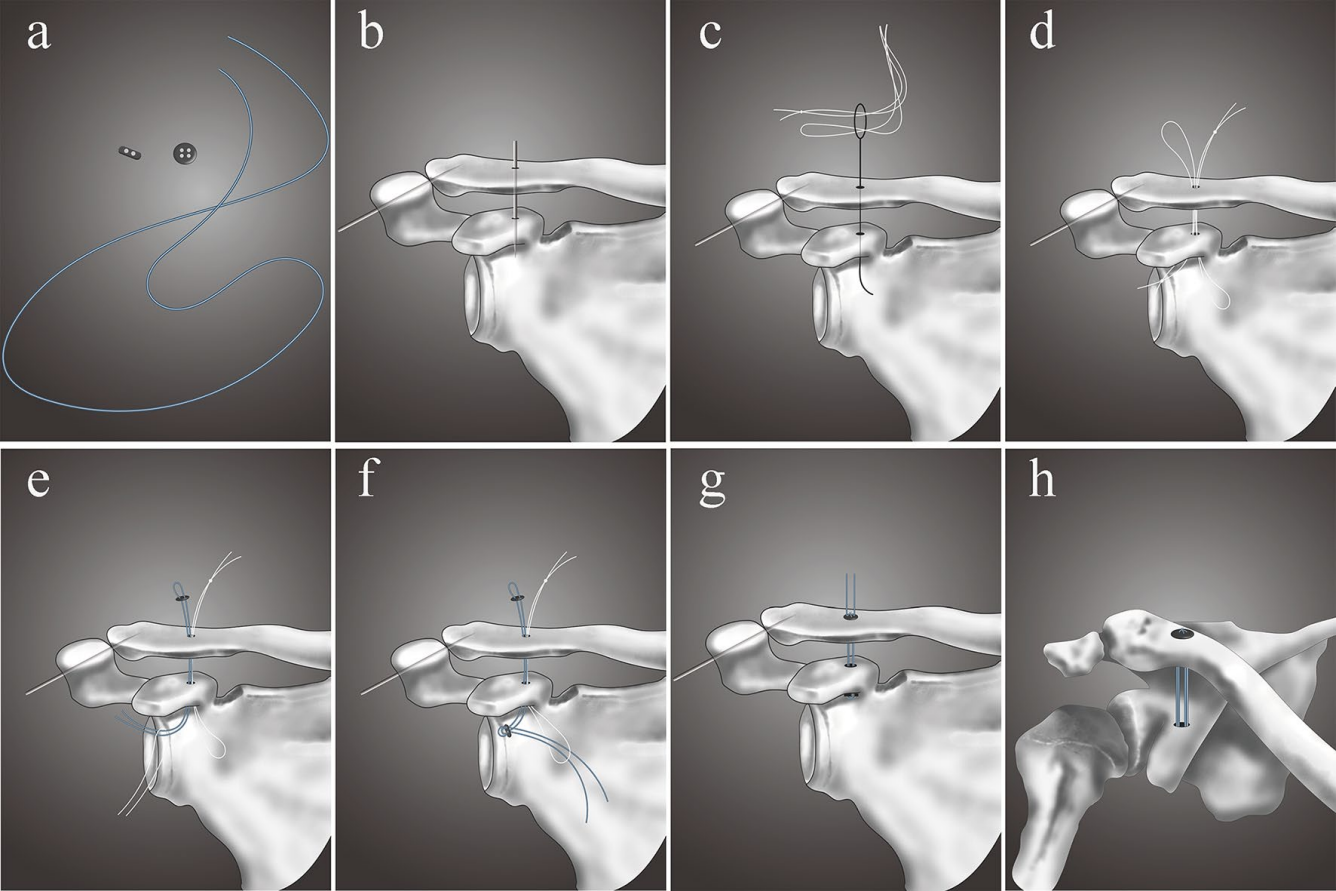
MITR modified operative procedures. (**a**) The TR system is dismantled. (**b**) A 2.5-mm Kirschner wire is introduced through the clavicle and coracoid. (**c,d**) The two white traction sutures are passed through the tunnels using a nitinol suture passing wire. (**e,f**) The TR system is introduced through the tunnels using the two white traction sutures and then reassembled. (**h**) Final frontal view of AC joint fixation with the TR system.

Despite the good postoperative results of the TR technique, complications have also been reported, probably owing to an initial malreduction, button displacement or inadequate healing of the disrupted ligaments^31^. Redislocation is a common complication of the TR technique^32^. In the present study, vertical redislocation with complete loss of reduction was identified in 1 patient (6.3%) in the MITR group. There are three causes of redislocation. The No. 5 suture of the TR system can sometimes be too weak, leading to redislocation. Thus, the sutures should be replaced by stronger sutures^33^. The 4.0 mm bone tunnel is a bit large; thus, the buttons can sometimes sink into the bone tunnel of the clavicle or coracoid^34^. Therefore, we designed a modified MITR procedure with a 2.5-mm bone tunnel replacing the 4.0-mm bone tunnel (Fig. 4). Finally, bone tunnel enlargement, which may be caused by residual anteroposterior instability, can lead to redislocation. Therefore, if the preoperative CT scan shows posterior displacement of the distal clavicle (type IV), additional AC fixation with two strong sutures is recommended.

The present study has several limitations that should be noted. The first limitation is the lack of preoperative scores for comparison. Second, this is a retrospective, nonrandomized control study. Third, athletic and work outcomes such as return to play or return to work may have added to the study. Fourth, the limited patient number and relatively short follow-up duration might weaken the strength of the results.

## Conclusion

Both the MITR technique and the HP technique were excellent choices for treating acute AC dislocations (type III and V). The MITR procedure and HP procedure showed similar radiological and clinical results in the present study. However, the MITR procedure provided a slightly lower tendency of pain and more convenience. Long-term follow-up is needed to investigate the clinical outcomes and radiological outcomes of both groups.
